# Comparison of detection percentage and morphology of myocardial bridge between conventional coronary angiography and coronary CT angiography

**DOI:** 10.15171/jcvtr.2019.34

**Published:** 2019-08-24

**Authors:** Seyed Hassan Eftekhar-Vaghefi, Somayeh Pourhoseini, Maryam Movahedi, Shohre Hooshmand, Mohammad Ali Ostovan, Pooyan Dehghani, Nikan Ostovan

**Affiliations:** ^1^Department of Anatomy, School of Medicine, Kerman University of Medical Sciences, Kerman, Iran; ^2^Department of Cardiology, School of Medicine, Shiraz University of Medical Sciences, Shiraz, Iran; ^3^Cardiovascular Research Center, Shiraz University of Medical Sciences, Shiraz, Iran

**Keywords:** Myocardial bridge, CT Angiography, Coronary Angiography

## Abstract

***Introduction:*** Myocardial bridge (MB) is a congenital anomaly in which a segment of a coronary artery is surrounded by myocardium. In our study, we want to use conventional coronary angiography (CCA) to describe morphologic characteristics of MB (unidentified or identified) in the patients with documented evidence of MB in coronary computed tomography angiography (CCTA).

***Methods:*** The present study was designed as cross-sectional and was conducted on 47 patients with documented evidence of MB in CCTA, who were referred to Nemazee and Faghihi hospitals for performing coronary angiography during a one year period. We compared the morphologic characteristics of tunneled segments, which were missed at CCA (unidentified), and the tunneled segments which were identified with CCA.

***Results:*** In sum, MB was found in 16 (34%) patients at CCA (identified), and it was not found in 31 (66%) patients (unidentified) based on compression sign. No significant correlation was found between the percentage of systolic compression and the length and depth of the tunneled segment in identified group (r=0.73, *P * = 0.18; r=1.09, *P * = 0.15; respectively). Degree of atherosclerotic plaque formation (diameter stenosis, percentage) (mean, 0.25 (25%) ±0.29; range, 0-0.98) of the tunneled segments in unidentified group was significantly more than the same degree (mean, 0.07 (7%) ±0.13; range, 0-0.41) of the identified group (*P * = 0.03). The measurement of the trapezoid area under the tunneled segment with this formula [(MB length+ intramyocardial segment) ×depth/2] had significant relation with systolic compression (r=0.304, P = 0.03) and defined the cut-off value of 250 mm^2^ as the value of significant difference in detecting myocardial bridging with CCA.

***Conclusion:*** Our results showed that in most of identified MBs in CCA the tunneled segment area was equal and more than 250 mm^2^. In addition, the degree of atherosclerotic plaque of the tunneled segments at CCA was significantly more in unidentified group.

## Introduction

Myocardial bridge (MB) is a coronary artery segment which is surrounded by myocardium and led to systolic compression.^1^ It is benign and has a more than 97%, 5-year survival rate.^1,2^ However it may cause life threating conditions such as, angina, acute coronary syndrome, myocardial ischemia, left ventricular dysfunction, and even sudden cardiac death due to hemodynamic changes.^2^ Also, high wall shear stress proximal to the MB induces the atherosclerotic plaque whereas the low wall shear stress within the tunneled part is found to be protective, so even in the absence of systolic compression there may be a prone focus for atherosclerotic changes.

Ferreira et al described two types of MB in left anterior descending (LAD) artery; superficial bridges that cross the artery vertically or at an acute angle toward the apex that comprise 75% of cases and deep bridges that are defined by muscle bundles arising from apical trabeculae of the right ventricle that cross the artery transversely, obliquely, or helically before inserting in the interventricular septum.^3^

The MB prevalence varies widely according to the methods used to investigate such an anomaly. In autopsy studies, it ranges from 15% to 85%^1,4^ while coronary angiography usually detects only 0.5%–12%. The prevalence is up to 40% by provocation tests.^5,6^ MB is routinely diagnosed by conventional coronary angiography (CCA) which indirectly indicates systolic compression of the tunneled segment and a focal change in vessel direction into the ventricle.^1,2^ It is hypothesized that deep type MB twists and constricts the tunneled segment causing marked systolic compression which may be absent in shallow type resulting in this large difference in MB detection rate.^3^

In Leschka and colleagues study on MB, the percentage of systolic compression correlated with MB depth, however the tunneled segment length had no correlation with the degree of systolic compression. In addition, more than 50% of the tunneled segments were missed with conventional angiography, so they suggested that diagnosis of MB by visual estimation at conventional angiography can only be made for segments with more than 20% systolic compression.^5^

This large difference between the prevalence of MB in autopsy and the depiction rate in CCA points to the absence of a gold standard imaging technique.^5^ MB is usually diagnosed by CCA which indirectly indicates systolic compression of the tunneled segment and a focal alteration in direction of the vessel into the ventricle.^3,6^ Although the current standard imaging modality for the diagnosis of myocardial bridging is coronary catheter,^7^ the diagnosis can also been made by intracoronary Doppler (ICD) and intravascular ultrasound (IVUS).^8,9^ All of the above, however, are invasive procedures. Development of coronary computed tomography angiography (CCTA) has helped to detect the whole course of coronary arteries and their adjacent myocardial structures, clearly.^10-12^ CCTA becomes available to be a reliable noninvasive method for detection of MB. Moreover, in recent studies CCTA has detected bridged coronary segments similar to the autopsy series.^13,14^

CCA underestimate the prevalence of MB as the investigators should rely on indirect signs in the assessment of vessel.^7^ Although systolic compression, the milking effect in addition to the step down–step up phenomenon are diagnostic signs, they are insensitive in MB shallow variants.^2,5,6,8^

Compared with CCA in CCTA tunneled segments and the surrounding tissue of coronary arteries could be assessed with no change in vessel course or even by only minimal or no systolic compression. Thus the discrepancy in the detection rate of MB between conventional angiography and CT supposed to be significantly correlated with its length, depth, and degree of systolic compression.^3,5^

It can be emphasized that many cases of bridging go unrecognized on angiography, such as involvement of the left circumflex and right coronary arteries.^15^

In a study of 100 patients using computed tomography (CT) angiography, MB of coronary arteries was found in 34 percent, but only approximately one-third of these showed systolic compression. In another study using CT angiography, MB was found to be a common anatomic variant.^16^

In our study we want to use CCA to describe morphologic characteristics of MB (unidentified or identified) in the patients with documented evidence of MB in CCTA. In the extent of our knowledge almost no specified study has been done on this field especially on characteristics of unidentified MB till now.

## Materials and Methods

We recruited 47 patients consecutively with documented evidence of MB in coronary CTA who referred to Nemazee and Faghihi hospitals, tertiary healthcare centers affiliated to Shiraz University of Medical Sciences (SUMS), for performing coronary angiography during 1-year period. Consecutive patients with confirmed LAD myocardial bridge on coronary CTA who underwent a coronary angiography for suspected coronary artery disease (presence of angina pectoris and at least one of the followings: ECG findings in favor of ischemia, abnormal exercise stress test or myocardial perfusion imaging or high atherosclerotic risk) were enrolled in the study. Exclusion criteria included history of previous coronary revascularization, history of previous contrast agent allergy, history of nephropathy (creatinine level >1.4 mg/dL), phosphodiesterase type 5 inhibitor consumption at least in 24 hour before coronary angiography and patients with poor technique imaging (CCTA, CAG).

In CCTA, reconstructed images were evaluated for all segments of coronary artery tree, which are described according to the current guidelines of the American Heart Association.^14^ Segments with a luminal diameter of less than 1.5 mm in their whole length were excluded.

MB was detected if a part of a coronary artery was completely restricted by myocardium. The location, length and depth of the tunneled segment were evaluated. The mean diameter of the tunneled segment at maximum depth was measured in one parallel and one perpendicular planes by electronic calipers. Measurements were done in the end-systolic and end-diastolic phases. The percentage of systolic compression was calculated by mean of these four measurements in end systole and end diastole.

Conventional coronary angiographies were performed by an expert interventional cardiologist with transfemoral approach, and with multiple different projections for each coronary artery after intracoronary injection of contrast agent. Angiography was routinely repeated after intracoronary injection of 200 μg nitroglycerin 2 times with one minute interval (if the blood pressure remained more than 90 mm Hg) and systolic compression, the length and of the tunneled segment in anteroposterior (AP) cranial view were compared before and after the administration of nitroglycerin. The angiograms were all recorded. Then, those were evaluated in consensus by our interventional cardiologists who were blinded to the results of CT coronary angiography. Analysis of conventional coronary angiographic data was done via offline quantitative coronary artery software in two steps:

First, all angiograms were reviewed by our blinded interventional cardiologists, and each vessel segment was visually analyzed for the presence of MB based on the following indirect signs: narrowing of systolic diameter; milking effect, explained as a diameter narrowing limited to a restricted vessel segment with extraction of contrast agent not interpretable by normal coronary artery flow; and/or the step down–step up phenomenon, explained as a localized change in direction of the vessel course into the ventricle.

Second, if MB was considered to be present, the grade of systolic diameter narrowing was measured in a way that CT measurements were determined. With comparing the luminal diameter within the tunneled segments in end systole and end diastole. Moreover, we measured the length, depth, and intramyocardial of the bridged segment using offline quantitative coronary artery software, and then reached product and area of the tunneled segment measures.

Statistical analyses were performed with the Statistical Package for Social Sciences version 19.0 (SPSS Inc., Chicago, IL, USA). The distribution of variables was assessed with Kolmogorov-Smirnov test. Quantitative variables with normal distribution were analyzed with a two-tailed Student’s *t* tests. Nonparametric variables were analyzed with the Wilcoxon test. Data are reported as means ± SD. A *P* value less than 0.05 was considered statistically significant.

## Results

Forty-seven patients with confirmed LAD myocardial bridge on coronary CTA were included in this study. Patient ages ranged from 44 to 84 years, with an average age of 58.97±8.75 years, and there were 22 (46.8%) male and 25 (53.2%) female.

MB was found in 16 (34%) patients at CCA (identified), and it was not found in 31 (66%) patients (unidentified) based on compression sign. After intracoronary nitroglycerin injection in 7 other patient MB was revealed, so totally 23 patients (48%) showed MB in CCA. In identified group, the mean systolic compression of the tunneled segment was 43.12% ± 24.41 ranged from 20 to 90%. No significant correlation was found between the percentage of systolic compression and the length and depth of the tunneled segment in identified patients (r=0.73, *P*=0.18; r=1.09, *P*=0.15; respectively). Although, correlation between the percentage of systolic compression and product of myocardial bridging was borderline and not statistically significant (r=0.48, *P*=0.05) correlation between the percentage of systolic compression and the trapezoid area under the myocardial bridging was statistically significant (r=0.304, *P*=0.03).

Baseline characteristics of both study groups were similar (Table 1). The mean age of the patients in identified group was found to be 59.75±10.51 years, and it was 58.58±7.86 years in unidentified group (*P*=0.66). Among the participants in identified group, there were 9 (56.2%) male and 7 (43.8%) female while there were 13 (43.8%) male and 18 (58.1%) female in unidentified group (*P*=0.35). The mean of ejection fraction (EF) was 59.43±3.03 in identified group, and it was 57.11±4.71 in unidentified group (*P*=0.08).

**Table 1:** Baseline characteristics of study patients

	**Identified group (n=16)**	**Unidentified group (n=31)**	***P*** ** value**
Age (y)	59.75±10.51	58.58±7.86	0.66
Gender			0.35
Male	9 (56.2%)	13 (41.9%)	
Female	7 (43.8%)	18 (58.1%)	
EF	59.43±3.03	57.11±4.71	0.08

EF: Ejection fraction

**Figure 1:**
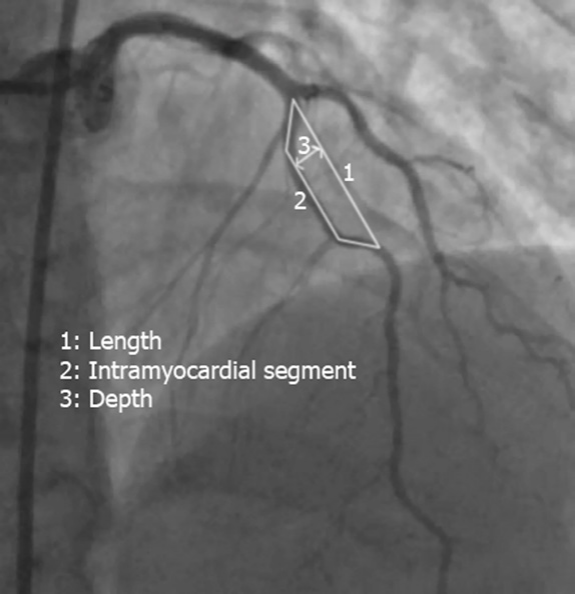
Trapezoid area calculation.

Degree of atherosclerotic plaque formation (mean, 0.07±0.13; range, 0-0.41) of the tunneled segments in unidentified group was significantly more than it (mean, 0.25±0.29; range, 0-0.98) in identified group (*P*=0.03). However, the length (mean, 34.56±11.54 mm; range, 15–50 mm) of the tunneled segments in identified group was slightly more than the length (mean, 30.77±10.28; range, 15-55 mm) of the tunneled segments missed at CCA (unidentified group), but difference was not significant (*P*=0.35). Similarly, the depth (mean, 11.37±8.24 mm; range, 4-30 mm) of the tunneled segments identified with CCA was not significantly difference with the depth (mean, 9.77±7.21; range, 4-35 mm) of the tunneled segments that were missed at CCA (unidentified group) (*P*=0.49). In addition, there were not significant differences between two study groups regarding tunneled segment product (length × depth), intramyocardial, angle of entry to myocardium, and angle of exit to myocardium (*P*=0.39, *P* = 0.14, *P* = 0.88, *P*=0.72, respectively). Also, we measured trapezoid area (Figure 1) under the tunneled segment with this formula [(length + intramyocardial) × depth/2]. However, the tunneled segment area (mean, 291.25±223.01 mm; range, 46-810 mm) in identified group was slightly more than it in unidentified group (mean, 219.65±199.53 mm; range, 47.5-927.5 mm), but difference was not significant (*P*=0.26). Table 2 represents comparing CCA estimations between two groups.

**Table 2:** Comparing CCA parameters of myocardial bridging between two groups

	**Identified group (n=16)**	**Unidentified group (n=31)**	**OR (95% CI)**	***P*** ** value**
Length of myocardial bridging (mm)	34.56±11.54	30.77±10.28	1.03 (0.97-1.09)	0.35
Depth of myocardial bridging (mm)	11.37±8.34	9.71±7.21	1.02(0.95-1.11)	0.49
Product of myocardial bridging (mm)	431±395	334±348	1.00(0.99-1.00)	0.39
Intramyocardial segment length (mm)	15.56±8.33	11.75±8.31	1.05(0.98-1.13)	0.14
Angle of entry to myocardium (degree)	39.87±7.22	40.32±10.94	0.99(0.93-1.06)	0.88
Angle of exit to myocardium (degree)	39.93±12.66	41.22±11.62	0.99(0.94-1.04)	0.72
Tunneled segment area (mm^2^)	291.25±223.01	219.65±199.53	1.00 (0.99-1.00)	0.26
Atherosclerotic plaque formation (Diameter stenosis percentage)	0.07 (7%) ±0.13	0.25 (25%) ±0.29	0.01(0.00-0.99)	0.03

In another analysis, we used Fisher’s exact test and stem-and-leaf plot to reach a cut-off value for detecting the tunneled segments at CCA. It was just found in tunneled segment area measurement. More than 70% of the patients with unidentified MB had tunneled segment area less than 250 mm^2^, while the most of the tunneled segments identified with CCA had segment area more and equal to 250 mm^2^. Actually, the cut-off value of 250 mm^2^ was defined as the value of significant difference in detecting MB with CCA (*P*=0.014) (Table 3).

**Table 3:** Comparing tunneled segment area in cut-off value 250 mm2

Cut-off value in tunneled segment area	Identified group (n=16)	Unidentified group (n=31)
<250 mm^2^	6 (37.5%)	23 (74.2%)
≥250mm2	10 (62.5%)	8 (25.8%)

## Discussion

Although MB historically was thought to be an incidental finding, it can cause some severe complications, such as acute myocardial infarction, different types of arrhythmia, exercise induced atrioventricular conduction block^17^ and sudden cardiac death.^18,19^

Prevalence of MB differs in pathological and angiographic studies. Autopsy studies report a mean frequency of MB of 25% (5%–86%).^20^ LAD is the most common involved coronary artery, however all major epicardial coronary arteries can be affected. The detection rate of MB in CCA studies is 0.5%–4.5%.^6^ This discrepancy between autopsy and conventional angiography points to the lack of an accurate gold standard imaging modality.

Ferreira et al hypothesized that systolic compression is minimal or even absent in the superficial type of MB and highly present in deep ones.^3^

Conventional angiography provides assessment limited to the vessel lumen in just one view per projection, and this limits the cardiologist to rely on indirect signs, so the prevalence of MB may be underestimated.^17^ Although milking effect and systolic compression are considered as diagnostic, these are insensitive in shallow forms of MB.^19^ In addition, the step down-step up phenomenon may be absent in superficial variants. The detection rate of MB in the present study was 34%, which is higher than previous angiography studies (0.5%–4.5%).^6^ The nearest detection rate to our study was 12% which was reported by Leschka et al.^5^ The probable reasons for this difference may be reviewing angiograms with the specific goal of finding the myocardial bridging, and including only selected patients with documented evidence of MB in CCTA.

There is controversy regarding the correlation between ischemic symptoms and the length of the tunneled segment or the degree of systolic compression.^3,20^ Some studies showed an elevated chance of ischemia and death in deep tunneled segments.^20^ As shown in our study, the percentage of systolic compression didn’t correlate with the length or depth of MB, while Leschka et al showed that the depth of the tunneled segment correlated with the percentage of systolic compression but not the length.^5^ It should be mentioned that they measured the depth and length of the tunneled segment by CCTA but these estimations were measured by CCA via offline quantitative coronary artery software in our study.

With regards to the incidence of atherosclerotic plaques in the tunneled segment, literature has depicted that the tunneled segment is affected rarely by atherosclerosis, in contrast to the epicardial segments. It has been hypothesized that the intramyocardial course of the coronary artery has a protective role in the development of atherosclerosis.^11^ Recently it has been shown that the segments proximal to the bridge are narrowed significantly, but the tunneled segment itself is free of atherosclerosis. Elevated wall shear stress proximal to the tunneled segment may be a predisposing factor for atherosclerosis.^20,21^ However, in our study, atherosclerotic lesions were seen about 40% of the tunneled segments, and degree of atherosclerotic plaque formation of the tunneled segments in unidentified group was significantly more than it in identified group (*P*=0.03). Enrolling the symptomatic patients who were suspicious ischemic coronary artery disease can be a probable reason for this difference with international view.

According to our results, all estimations of tunneled segments (including, length, depth, product, and area) in identified MBs with CCA were slightly more than those in unidentified MBs with CCA, but these differences were not significant. In contrast a previous study, demonstrated both depth and length in identified group are significantly greater than those in unidentified group.^5^ In their study all estimations of tunneled segment were done based on CCTA, but we measured all of them with CCA. Moreover, the small number of patients in our study decreased the power of study to demonstrate precisely these differences.

As new findings, our study demonstrated that there is a trapezoid area under the bridge segment with a significant correlation between this trapezoid area and systolic compression of the bridge segment. Besides, the cut-off value of 250 mm^2^ was defined as the value of significant difference in recognition of MB with CCA. More than 70% of the MB that were missed at CCA had tunneled segment area less than 250 mm^2^, while the most of the tunneled segments identified with CCA had segment area equal and more than 250 mm^2^. Undoubtedly, our finding is difficult to interpret and could probably result from the small number of patients; therefore, higher sample sizes are suggested in future studies.

This study had a number limiting factors, of which the small number of patients was the most important. An additional limitation is that we only compared estimations of tunneled segments based on CCA measurements. Therefore, larger prospective studies including CCTA measurements are needed to determine all aspects of this matter.

## Conclusion

To our knowledge, this is the first study which especially investigat­ing and comparing morphologic characteristics of identified and unidentified MBs based on CCA estimations. In conclusion, our results show that the most identified MBs in CCA had the tunneled segments area equal and more than 250 mm^2^. In addition, degree of atherosclerotic plaque formation of the tunneled segments that were missed at CCA was significantly more than it in identified group.

## Competing interests

None.

## Ethical approval

The study protocol was approved by the institutional review board (IRB) of SUMS and we obtained ethics approval from the local ethics committee before the study was commenced (Ethical Code: IR.sums.med.rec.1396.s103). All the participants signed the written informed consent.

## Acknowledgments

This article was extracted from the thesis written by Maryam Movahedi for the degree of cardiology specialty and was financed and supported by Research Vice-chancellor of Shiraz University of Medical Sciences (grant No. 9830).
